# Comparative Phenotypic and Transcriptomic Analysis Reveals Distinct Yet Partially Convergent Mechanisms of Daphnetin and 6-Methylcoumarin Against Eucalyptus-Derived *Ralstonia pseudosolanacearum*

**DOI:** 10.3390/microorganisms14081799

**Published:** 2026-08-14

**Authors:** Han Xue, Ning Jiang, Yong Li

**Affiliations:** Key Laboratory of Forest Protection of National Forestry and Grassland Administration, Ecology and Nature Conservation Institute, Chinese Academy of Forestry, Beijing 100091, China; xhsynlx@163.com (H.X.); n.jiang@caf.ac.cn (N.J.)

**Keywords:** *Ralstonia pseudosolanacearum*, daphnetin, 6-methylcoumarin, RNA-seq, type III secretion system, extracellular polysaccharides

## Abstract

Eucalyptus bacterial wilt in China, caused predominantly by *Ralstonia pseudosolanacearum*, severely threatens Eucalyptus plantation management in China and demands plant-derived alternatives to conventional bactericides. This study evaluated the in vitro antibacterial activities and underlying molecular mechanisms of two differentially substituted coumarin derivatives, daphnetin (DAP) and 6-methylcoumarin (MC), against a eucalyptus-derived *R. pseudosolanacearum* strain. The minimum inhibitory concentration (MIC) and minimum bactericidal concentration (MBC) were determined using broth microdilution assays. Quantitative phenotypic assays were conducted to evaluate the effects of sub-lethal concentrations on biofilm formation, swimming motility, and extracellular polysaccharide (EPS) accumulation. Furthermore, high-throughput RNA sequencing integrated with GO and KEGG enrichment analyses was employed to characterize the genome-wide transcriptomic responses. Antimicrobial susceptibility testing demonstrated that DAP possessed superior bactericidal efficacy, yielding lower MIC (62.5 μg/mL) and MBC (250 μg/mL) values than MC. Conversely, sub-lethal MC exhibited a more pronounced, early-stage suppression of flagellum-dependent swimming motility and biofilm maturation. Both compounds consistently attenuated EPS production. Transcriptomic analysis revealed distinct yet partially convergent regulatory networks: DAP primarily exerted metabolic strangulation by downregulating genes governing aerobic respiratory chains and peripheral carbon/nitrogen pathways, whereas MC targeted collective behavior and active pathogenesis, systematically downregulating the expression of genes associated with flagellar assembly, quorum sensing, and Type III and VI secretion systems. Notably, both pathways converged downstream to repress the master virulence regulator *xpsR* and the *epsA-P* operon. These findings provide valuable insights into the potential molecular pathways affected by DAP and MC, offering a useful reference for future exploration of botanical formulations for ecologically responsible forest disease control.

## 1. Introduction

The *Ralstonia solanacearum* species complex (RSSC), encompassing several closely related soil-borne phytopathogenic species, is the causative agent of bacterial wilt in a wide range of economically important crops and forest trees. Members of the RSSC are characterized by their high persistence in soil and water, broad host range, and remarkable genetic diversity, making this group one of the most destructive bacterial plant pathogens worldwide. The infection process involves a multifactorial pathogenic mechanism—including chemotaxis, motility, host adhesion, cell wall degradation, and the massive production of extracellular polysaccharides (EPS) and biofilm formation—which collectively drive vascular collapse, resulting in symptoms ranging from partial withering and growth reduction to severe vascular decline or plant death [1,2,3]. RSSC encompasses several closely related species, exhibiting pronounced genomic diversity, extensive physiological differentiation, and high population structuring [4,5]. Strains within the RSSC display marked variation in host range, geographic prevalence, and core biological properties—including pathogenicity and carbon source utilization profiles. Under modern polyphasic taxonomy, the RSSC has been reclassified into three distinct species: *R. solanacearum* (Phylotype II), *R. pseudosolanacearum* (Phylotypes I and III), and *R. syzygii* (Phylotype IV) [6]. Among these, *R. pseudosolanacearum* (Phylotype I) and *R. solanacearum* (Phylotype II) are the primary etiological agents of eucalyptus bacterial wilt, displaying distinct geographic prevalence: *R. pseudosolanacearum* predominates in Asia and Africa (including China), whereas *R. solanacearum* is mainly associated with eucalyptus wilt in South America [7]. In China, *R. pseudosolanacearum* is the predominant pathogen, primarily affecting multiple provinces in South China, including major regions such as Guangdong, Guangxi, and Hainan [8].

Many strategies have been developed to manage bacterial wilt, including biological, chemical, cultural, and integrated management approaches [9,10]. However, effective control of this disease remains particularly challenging due to the pathogen’s prolonged soil survival, exceptionally broad host range, and protected vascular colonization. Traditional chemical interventions—including the use of copper hydroxide, zinc thiazole, thiediazole-copper, streptomycin, and soil fumigants like dazomet—have demonstrated a certain degree of field efficacy [11,12,13,14]. However, the frequent and excessive application of these synthetic agents often precipitates deleterious environmental consequences, including the emergence of pesticide-resistant strains, disruption of soil microbial ecology, and broader environmental safety concerns [15,16,17]. While breeding for genetic resistance is widely considered the most cost-efficient and eco-compatible strategy, resistance-related genetic improvement frequently entails trade-offs with yield-associated traits and product quality [18]. Consequently, botanical control—particularly the utilization of plant-derived extracts and compounds—has emerged as a promising alternative [19]. These botanical compounds not only inhibit soil-borne pathogens but also frequently promote plant growth [20]. Recent studies have identified several bioactive agents capable of suppressing *R. solanacearum*, including broad chemical classes like coumarins [21], flavonoids [22], terpenoids [23], phenols [24], and volatile mixtures such as essential oils from citronella and eucalyptus [25].

Notably, Coumarins and their derivatives are widespread plant secondary metabolites synthesized via the phenylpropanoid pathway as a defense response [26]. These compounds exhibit broad-spectrum antimicrobial activity, making them attractive structural templates or lead compounds for future eco-friendly pesticide research [27,28]. Extensive research indicates that coumarin derivatives reduce *R. solanacearum* virulence primarily by repressing biofilm formation and key virulence genes, though their specific cellular targets vary by chemical structure. Among hydroxycoumarins, DAP mechanically disrupts cell membranes [29], inhibits LPS biosynthesis, reduces EPS via *xpsR* and *epsS* downregulation, and affects T3SS and T4SS genes [21]; umbelliferone similarly targets biofilm formation while repressing T3SS and six representative effector genes [30]. Among methylcoumarins, MC uniquely targets bacterial growth by suppressing *ftsZ* to block cytokinesis and induce filamentation [31], whereas 7-methylcoumarin restricts biofilm development and represses key virulence genes including *epsE*, *hrpG*, and *popA* [32]. Importantly, our preliminary metabolomic analysis of eucalyptus xylem sap revealed that both DAP and MC—representing hydroxy- and methyl-substituted coumarin scaffolds, respectively—were significantly up-regulated upon *R. pseudosolanacearum* infection (unpublished data). These results supported the hypothesis that DAP and MC serve as key host-derived defense metabolites during eucalyptus-pathogen interactions. Nevertheless, the precise antibacterial modes of action and underlying molecular mechanisms of these two distinct chemical scaffolds against *Ralstonia* species remain to be fully elucidated. Furthermore, current research on coumarin-based management of bacterial wilt predominantly focuses on *R. solanacearum* infecting tobacco and tomato [30,33]. Given that *R. pseudosolanacearum*—specifically the strain associated with eucalyptus bacterial wilt—possesses unique host adaptation, metabolic profiles, and transcriptional regulatory networks [8], the transcriptomic response profiles and underlying molecular mechanisms of this specific strain under coumarin treatment remain unknown. Uncovering these systemic differences directly facilitates the design of high-efficacy, targeted biopesticides for forest management.

To address this, the present study evaluated the in vitro antibacterial activity of DAP and MC against the eucalyptus-derived *R. pseudosolanacearum*, and investigated their impacts on biofilm formation, EPS production, and motility. Additionally, RNA sequencing (RNA-seq) was employed to identify key differentially expressed genes (DEGs) involved in cellular functions and pathogenic processes. Therefore, this study aims to investigate the transcriptomic responses and molecular targets of these coumarin derivatives in *R. pseudosolanacearum*, thereby clarifying their mode of action against this eucalyptus-infecting pathogen.

## 2. Materials and Methods

### 2.1. Bacterial Strain and Chemicals

The *R. pseudosolanacearum* strain XM6 used in this study was isolated from the diseased stem tissues of infected *Eucalyptus grandis* in Qinzhou City, Guangxi Province, China, and has been deposited at the China Forestry Culture Collection Center with the accession number CFCC 17426. Species-level identification of this strain was previously established through a polyphasic approach comprising 16S rRNA gene sequencing, whole-genome phylogenetic analysis, and Average Nucleotide Identity comparison against reference genomes, which unambiguously confirmed its taxonomy as *R. pseudosolanacearum* [8]. DAP and MC (HPLC purity ≥ 98%) were obtained from Shanghai Yuanye Bio-Technology Co., Ltd. (Shanghai, China) and individually solubilized in dimethyl sulfoxide (DMSO, Macklin Biochemical Technology Co., Ltd., Shanghai, China), while streptomycin sulphate was obtained from Macklin Biochemical Technology Co., Ltd. (Shanghai, China) and solubilized in sterile double-distilled water.

### 2.2. Determination of MIC and MBC

The minimum inhibitory concentration (MIC) and minimum bactericidal concentration (MBC) of coumarin derivative against *R. pseudosolanacearum* were determined using the broth microdilution method in sterile 96-well polystyrene plates, following previously described protocols [34]. An overnight culture of *R. pseudosolanacearum* was adjusted to an OD_600_ of 1.0 (corresponding to approximately 1 × 10^8^ cfu/mL, established using a standardized OD_600_ calibration curve on a spectrophotometer and validated via serial dilution plate counts). Each well of a sterile 96-well plate contained a total working volume of 200 μL, comprising 198 µL of BG medium (1% bacto peptone, 0.1% casamino acids, 0.1% yeast extract, and 0.5% glucose) [35], 1 μL of the prepared bacterial suspension, and 1 μL of the antimicrobial solution, yielding a final cell density of approximately 5 × 10^5^ cfu/mL. The stock antimicrobial solutions were added to achieve final working concentrations ranging from 31.25 to 2000 μg (specifically: 31.25, 62.5, 125, 250, 500, 1000, 2000 μg/mL). Streptomycin sulphate was included as a positive control under identical conditions [36]. Growth was measured as OD_600_ using a Microplate reader (Multiskan FC, Thermo Scientific, Waltham, MA, USA) following static incubation. The MIC was defined as the minimum concentration of the coumarin compounds necessary to prevent bacterial growth after 48 h of incubation at 30 °C. The growth limit was deemed to be a value of 0.2 for OD_600_. To determine the MBC, 10 µL aliquots from each well were subcultured onto BG medium agar plates and incubated at 30 °C for 48 h. The MBC was identified as the lowest concentration that yielded no colony formation. Each experiment was conducted with three independent biological replicates using separate bacterial cultures. For each biological replicate, treatments were performed in triplicate (technical replicates). The data from technical replicates within each plate were averaged prior to statistical analysis, resulting in a final sample size of *n* = 3 for each treatment group.

### 2.3. Biofilm Biomass Assay

Biofilm biomass was quantified using a microtiter plate assay [37]. A standardized culture of *R. pseudosolanacearum* (OD_600_ = 1.0) was diluted 1:200 in BG medium and 199 μL mixture was inoculated into 96-well plates (final density 5 × 10^5^ cfu/mL) supplemented with 1 μL either DAP or MC at final concentrations of 20, 40, 80, or 160 μg/mL. Medium supplemented with 0.4% (*v*/*v*) DMSO (Macklin Biochemical Technology Co., Ltd., Shanghai, China) treatments served as controls. After 12, 24, and 36 h incubation at 30 °C, wells were gently washed with 200 μL of sterile phosphate-buffered saline (PBS, pH 7.4, Macklin Biochemical Technology Co., Ltd., Shanghai, China). The total adhered biomass (comprising cells and extracellular matrix) was stained by adding 200 μL of 0.1% (*w*/*v*) crystal violet solution (Macklin Biochemical Technology Co., Ltd., Shanghai, China) per well and incubating at room temperature for 15 min. Excess dye was removed, and the wells were rinsed three times with sterile distilled water and air-dried. The bound crystal violet was solubilized in 200 μL of 95% (*v*/*v*) ethanol for 20 min with gentle shaking, and total biomass was quantified by measuring the absorbance at 530 nm (OD_530_) using a microplate reader (Multiskan FC, Thermo Scientific, Waltham, MA, USA). Independent parallel 96-well plates were prepared for each designated duration. Each experiment was conducted with three independent biological replicates, with three technical replicate wells per treatment within each plate (*n* = 3).

### 2.4. Motility Assay

Swimming motility of *R. pseudosolanacearum* was evaluated on semi-solid agar as previously described [3], with minor modifications. DAP or MC was incorporated into BG medium with 0.3% agar to achieve final concentrations of 20, 40, 80, or 160 μg/mL. BG medium supplemented with an equal volume of DMSO served as the control. A 5 µL aliquot of bacterial suspension (OD_600_ = 0.1) was spot-inoculated at the center of each plate. After incubation at 30 °C for 24 h and 48 h, motility was quantified by measuring the diameter of the expansion zone. All assays were conducted in triplicate across three independent biological replicates.

### 2.5. EPS Quantification

To evaluate the effect on EPS accumulation, the hexosamine content associated with the crude EPS fraction of *R. pseudosolanacearum*—a major functional constituent reflecting the level of pathogenic EPS I—was determined using the Elson–Morgan assay, following the protocol described by Peyraud et al. [38]. Overnight bacterial cultures were harvested by centrifugation (6000× *g*, 5 min, 4 °C), washed twice with sterile PBS to remove residual polymers, and resuspended in fresh BG medium. The standardized bacterial suspension (OD_600_ = 0.20) was supplemented with DAP or MC at final concentrations of 20, 40, 80, or 160 μg/mL, or 0.4% (*v*/*v*) DMSO as a vehicle control. Cultures were incubated statically at 30 °C for 12 h, 24 h, and 36 h with separate parallel culture tubes prepared for each independent time point. At each indicated time point, cell-free supernatants were obtained by filtration through 0.22 μm membrane filters (Macklin Biochemical Technology Co., Ltd., Shanghai, China). Crude EPS was precipitated from 2.5 mL aliquots of the filtrate by adding NaCl to a final concentration of 0.1 M, followed by the dropwise addition of four volumes of pre-chilled (−20 °C) acetone. After overnight incubation at 4 °C (16 h), the precipitated polymers were pelleted by centrifugation (12,000× *g*, 20 min, 4 °C). The resulting pellets were resuspended in 200 μL of deionized water and heated at 65 °C for 15 min with continuous vortexing until visually homogeneous (free of visible aggregates). Insoluble debris was subsequently removed by a second centrifugation step (12,000× *g*, 10 min, 4 °C), and the clarified supernatant was subjected to acid hydrolysis with 6 M HCl at 110 °C for 30 min to liberate hexosamine monomers. Following neutralization and desalting, absorbance was measured spectrophotometrically at 530 nm, and hexosamine concentrations were quantified against a calibration curve generated using N-acetyl-D-galactosamine (Macklin Biochemical Technology Co., Ltd., Shanghai, China) as the reference standard. All assays were conducted in triplicate across three independent biological replicates.

### 2.6. Transcriptomic Analysis and Mechanism Study

*R. pseudosolanacearum* was cultured in BG medium supplemented with coumarin derivatives (DAP or MC) at their respective sub-inhibitory concentrations (1/2× MIC, corresponding to 37.5 μg/mL for DAP and 75 μg/mL for MC), or an equivalent volume of DMSO as a vehicle control. Cultures were incubated at 30 °C for 24 h with intermittent gentle shaking (slow orbital agitation for 20 s every 6 h). Under these conditions, both compounds achieved a comparable level of growth inhibition (~45–50% reduction in OD_600_ relative to the control) at the 24 h harvest time point, corresponding to the late-logarithmic to early-stationary growth phase. Cells were harvested via centrifugation at 8000× *g* for 5 min at 4 °C, immediately flash-frozen in liquid nitrogen, and stored at −80 °C. All treatments were performed in triplicate with independent biological replicates. Total RNA was extracted using the RNA Kit for Bacteria (Omega Bio-tek, Inc., Norcross, GA, USA) according to the manufacturer’s protocol, accompanied by on-column DNase I treatment to eliminate genomic DNA contamination. RNA integrity and concentration were evaluated using 1% agarose gel electrophoresis, a NanoDrop 2000 spectrophotometer (Thermo Fisher Scientific, Waltham, MA, USA), and an Agilent 2100 Bioanalyzer (Agilent Technologies, Santa Clara, CA, USA); only samples with RNA Integrity Number (RIN) > 8.0 and A_260_/A_280_ ratios between 1.9 and 2.1 were used for library construction. Transcriptome sequencing was subsequently performed by Novogene Co., Ltd. (Beijing, China). The clean reads were mapped to the reference genome of *R. pseudosolanacearum* LMG 9673 (NCBI assembly accession: GCA_024925465.1). Differential expression analysis was performed using DESeq2 (v1.42.0), with differentially expressed genes (DEGs) defined by an absolute | log2 fold change | > 1 and a Benjamini–Hochberg adjusted *p* < 0.05. Functional enrichment of DEGs was characterized via Gene Ontology (GO) and Kyoto Encyclopedia of Genes and Genomes (KEGG) analyses using the clusterProfiler (v4.10.0) package in R (v4.3.1) [39,40]. This approach allowed for the identification of significantly enriched metabolic pathways in *R. pseudosolanacearum*, thereby facilitating the screening of key candidate genes involved in these primary pathways.

### 2.7. Statistical Analysis

All experimental data were expressed as the mean ± standard deviation (SD) derived from at least three independent biological replicates (*n* = 3). Data normality and homogeneity of variance were verified using the Shapiro-Wilk test and Levene’s test, respectively. Statistical analysis was performed using the SPSS software package (version 28; SPSS Inc., Chicago, IL, USA). Pairwise comparisons between two groups were executed via Student’s *t*-test, whereas multiple comparisons among three or more groups were analyzed using one-way analysis of variance (ANOVA) followed by Duncan’s multiple range test. A *p*-value < 0.05 was considered statistically significant.

## 3. Results

### 3.1. MIC and MBC of DAP and MC Against R. pseudosolanacearum

**Figure 1:**
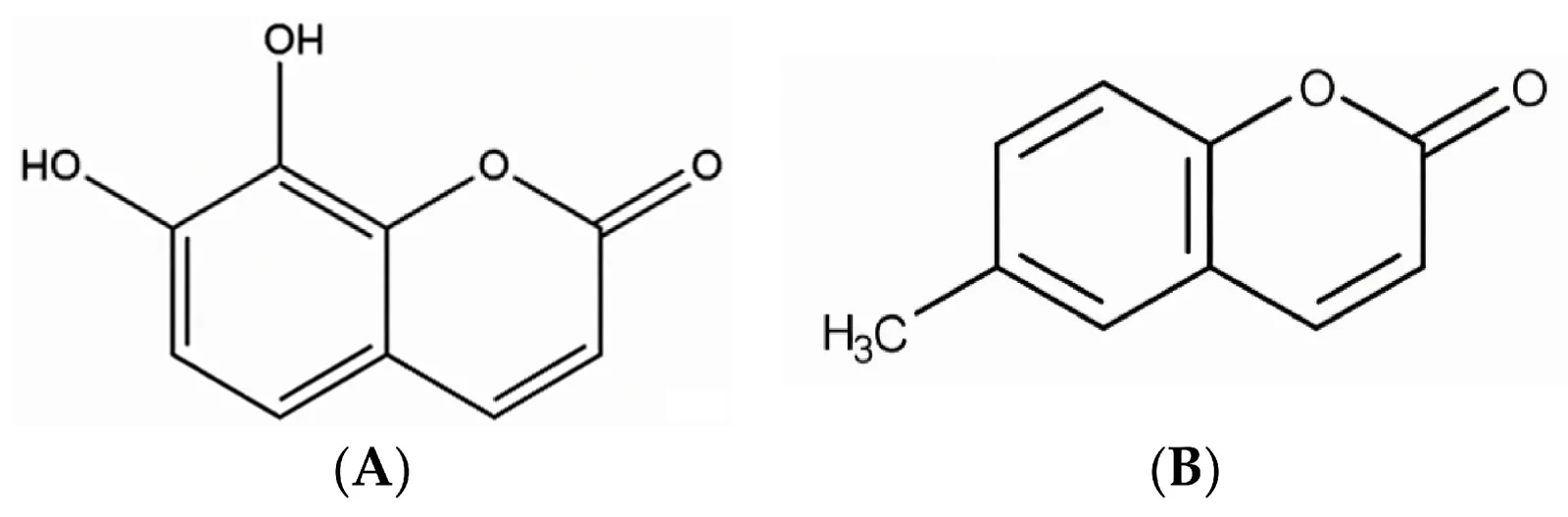
Chemical structures of daphnetin (DAP) and 6-methylcoumarin (MC). (**A**) DAP: 7,8-dihydroxy-2H-chromen-2-one, C_9_H_6_O_4_; (**B**) MC: 6-methyl-2H-chromen-2-one, C_10_H_8_O_2_.

**Figure 2:**
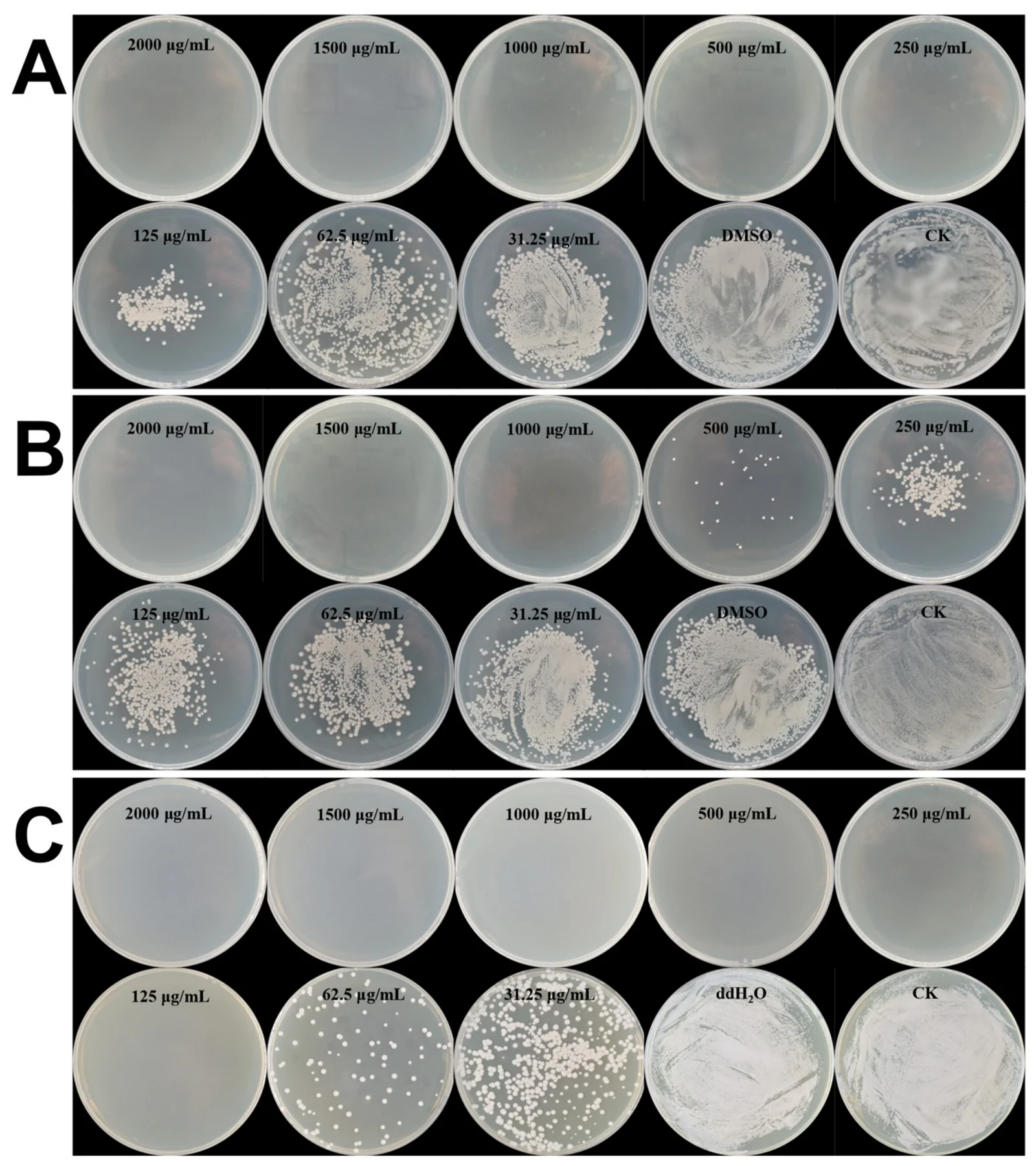
MBC of DAP (**A**), MC (**B**) and Streptomycin (**C**) against *R. pseudosolanacearum*.

The structures of DAP and MC are shown in Figure 1. Antimicrobial susceptibility testing demonstrated that DAP possessed superior bactericidal efficacy, yielding a MIC of 62.5 μg/mL, which represents a two-fold enhancement in inhibitory activity over MC (MIC = 125 μg/mL). Streptomycin sulphate, used as the positive control, exhibited strong antibacterial activity with a MIC of 31.2 μg/mL. MBC values were determined as the lowest concentrations that prevented colony formation following subculture on BG agar, thereby confirming the complete eradication of viable bacteria. Consistent with the MIC trends, DAP also demonstrated a much lower MBC (250 μg/mL), while the corresponding MBCs for MC were 1000 μg/mL. Meanwhile, the positive control streptomycin sulphate achieved complete bacterial elimination with an MBC of 125 μg/mL (Figure 2). The exact cell viability (CFU/mL) at each compound concentration is detailed in the Table S1.

### 3.2. Effects of DAP and MC on R. pseudosolanacearum Swimming Motility

**Figure 3:**
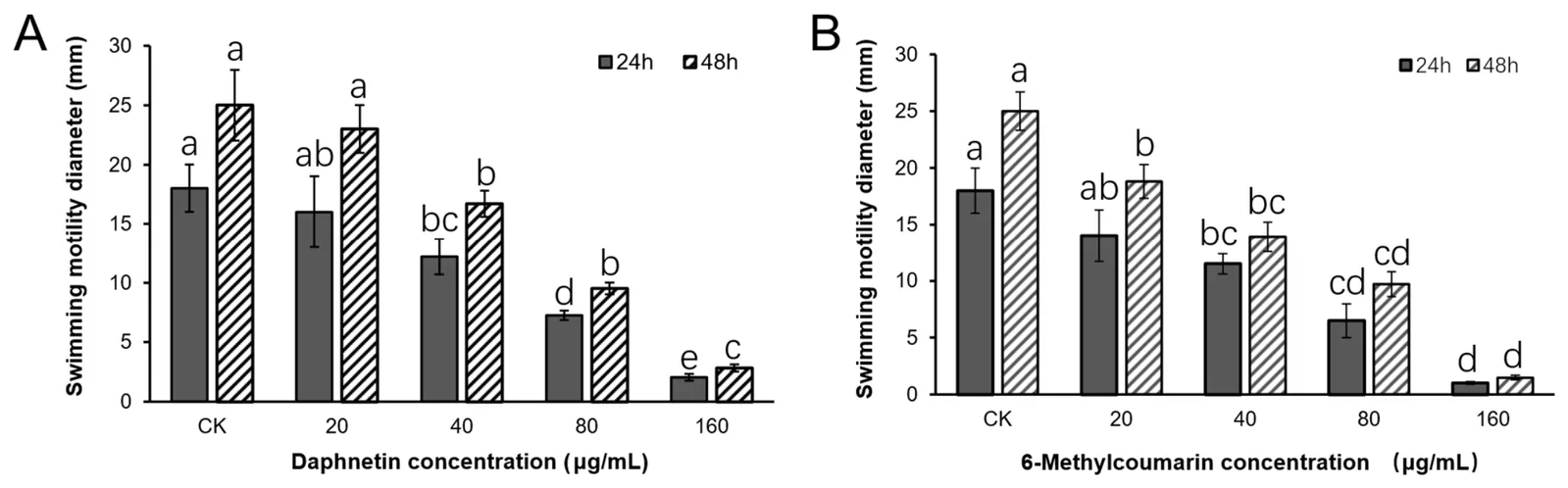
Effects of DAP and MC concentration on the motility of *R. pseudosolanacearum*. (**A**) Treatment with DAP at 24 h and 48 h, (**B**) Treatment with MC at 24 h and 48 h. Data are presented as mean ± SD (*n* = 3). Different lowercase letters above the bars indicate statistically significant differences among treatments within the same incubation time point (*p* < 0.05, analyzed by one-way ANOVA followed by Duncan’s multiple range test).

In this study, the inhibitory effects of DAP and MC on *R. pseudosolanacearum* swimming motility were systematically evaluated using a plate assay. Both compounds significantly suppressed bacterial motility in a concentration-dependent manner. Following 48 h of incubation, DAP and MC treatment across a concentration range of 20 to 160 μg/mL reduced the swimming motility diameter relative to the control by 8.09–88.64% and 26.07–94.32%, respectively (Figure 3). To directly compare the efficacy of the two compounds, a joint statistical analysis was conducted at each equivalent concentration (Table S2). The pairwise comparison demonstrated that MC exerted a significantly stronger inhibitory effect on swimming diameter than DAP at lower concentrations of 20 μg/mL (*p* = 0.0252) and 40 μg/mL (*p* = 0.0143). However, at higher concentrations (80 and 160 μg/mL), the inhibitory effects of DAP and MC showed no statistically significant differences (*p* > 0.05). These findings indicate that MC exhibits superior inhibitory potency against *R. pseudosolanacearum* swimming motility specifically at lower concentrations.

### 3.3. Effects of DAP and MC on R. pseudosolanacearum Biofilm Biomass

**Figure 4:**
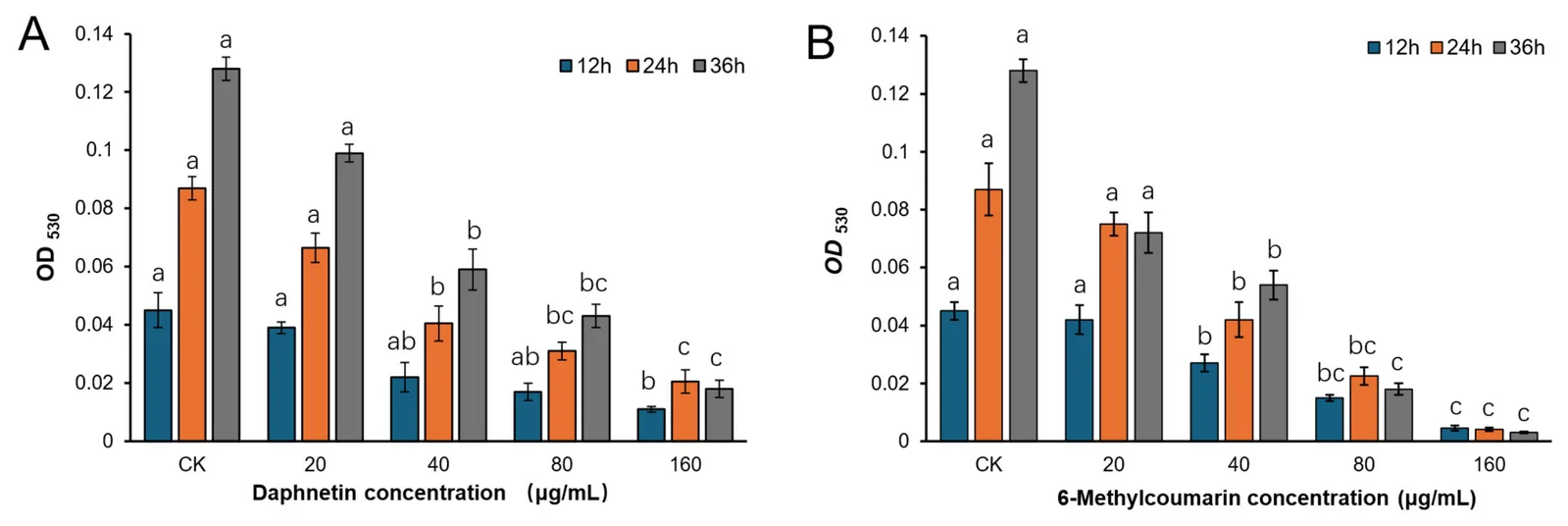
Effects of DAP and MC concentration on the biofilm biomass of *R. pseudosolanacearum*. (**A**) Treatment with DAP at 12 h, 24 h and 36 h, (**B**) Treatment with MC at 12 h, 24 h and 36 h. Data are presented as mean ± SD (*n* = 3). Different lowercase letters above the bars indicate statistically significant differences among treatments within the same incubation time point (*p* < 0.05, analyzed by one-way ANOVA followed by Duncan’s multiple range test).

Both DAP and MC impaired the biofilm formation of *R. pseudosolanacearum* in a distinct concentration-dependent manner across all tested time points (12, 24, and 36 h) (Figure 4A,B). In the control group (CK), biofilm biomass accumulated progressively over time, reaching values of 0.045 ± 0.003, 0.87 ± 0.009, and a peak of 1.28 ± 0.04 at 12, 24, and 36 h of incubation, respectively. Both compounds disrupted this accumulation over time and significantly suppressed biofilm accumulation relative to the control at 40 μg/mL and above (*p* < 0.05). To rigorously evaluate their inhibitory efficacies, a joint statistical analysis was performed on the 36 h biofilm data (Table S3). Following 36 h of exposure, MC demonstrated significantly greater suppression of biofilm formation than DAP at 20 μg/mL (*p* = 0.0247), achieving a 43.75% reduction compared to 22.66% for DAP. Although both compounds exhibited comparable inhibitory activity at 40 μg/mL (*p* = 0.2918), MC again exerted markedly superior inhibition at 80 μg/mL (*p* = 0.0006) and 160 μg/mL (*p* = 0.0003), achieving an inhibition rate of 97.66% compared to 85.93% for DAP at 160 μg/mL. These findings confirm that MC exhibits statistically significantly higher potency than DAP in suppressing *R. pseudosolanacearum* biofilm biomass.

### 3.4. Effects of DAP and MC on R. pseudosolanacearum EPS Production

**Figure 5:**
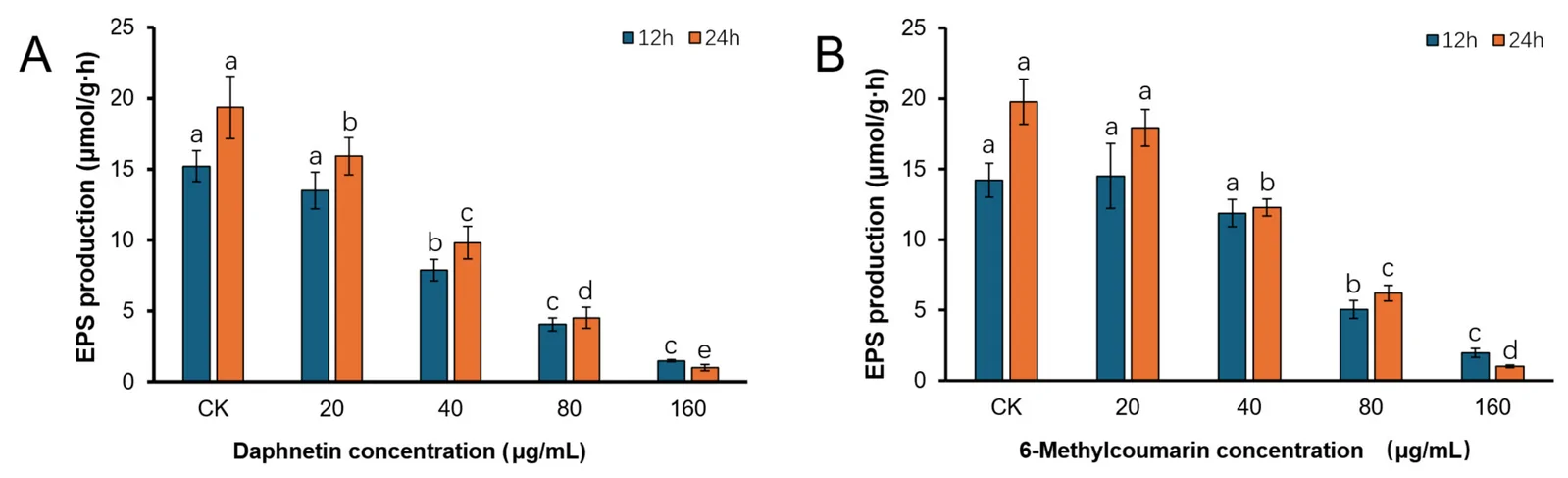
Effects of DAP and MC concentration on EPS production of *R. pseudosolanacearum*. (**A**) Treatment with DAP at 24 h and 48 h, (**B**) Treatment with MC at 12 h and 24 h. Data are presented as mean ± SD (*n* = 3). Different lowercase letters above the bars indicate statistically significant differences among treatments within the same incubation time point (*p* < 0.05, analyzed by one-way ANOVA followed by Duncan’s multiple range test).

The EPS production of *R. pseudosolanacearum* was decreased to varying degrees after treatment with DAP (Figure 5A) and MC (Figure 5B) at different concentrations. Specifically, at a low concentration of 20 μg/mL, DAP treatment triggered a statistically significant decrease in EPS yield to 15.92 ± 1.31 μmol/g·h, whereas the value for MC (17.92 ± 1.28) remains statistically indistinguishable from the CK. Direct pairwise comparisons between the two compounds further revealed that DAP exhibited significantly greater anti-EPS activity than MC across concentrations from 20 to 80 μg/mL (*p* < 0.05; Table S4). Notably, when the concentration was escalated to 160 μg/mL, both compounds nearly abolished EPS accumulation, cutting production down to approximately 1.0 μmol/g·h with no significant difference between treatments (*p* = 0.2362). Collectively, these findings indicate that while both compounds possess potent anti-EPS production at high doses, DAP exerts a significantly stronger inhibitory effect at low-to-moderate concentrations.

### 3.5. Transcriptomic Responses of R. pseudosolanacearum to DAP and MC

**Figure 6:**
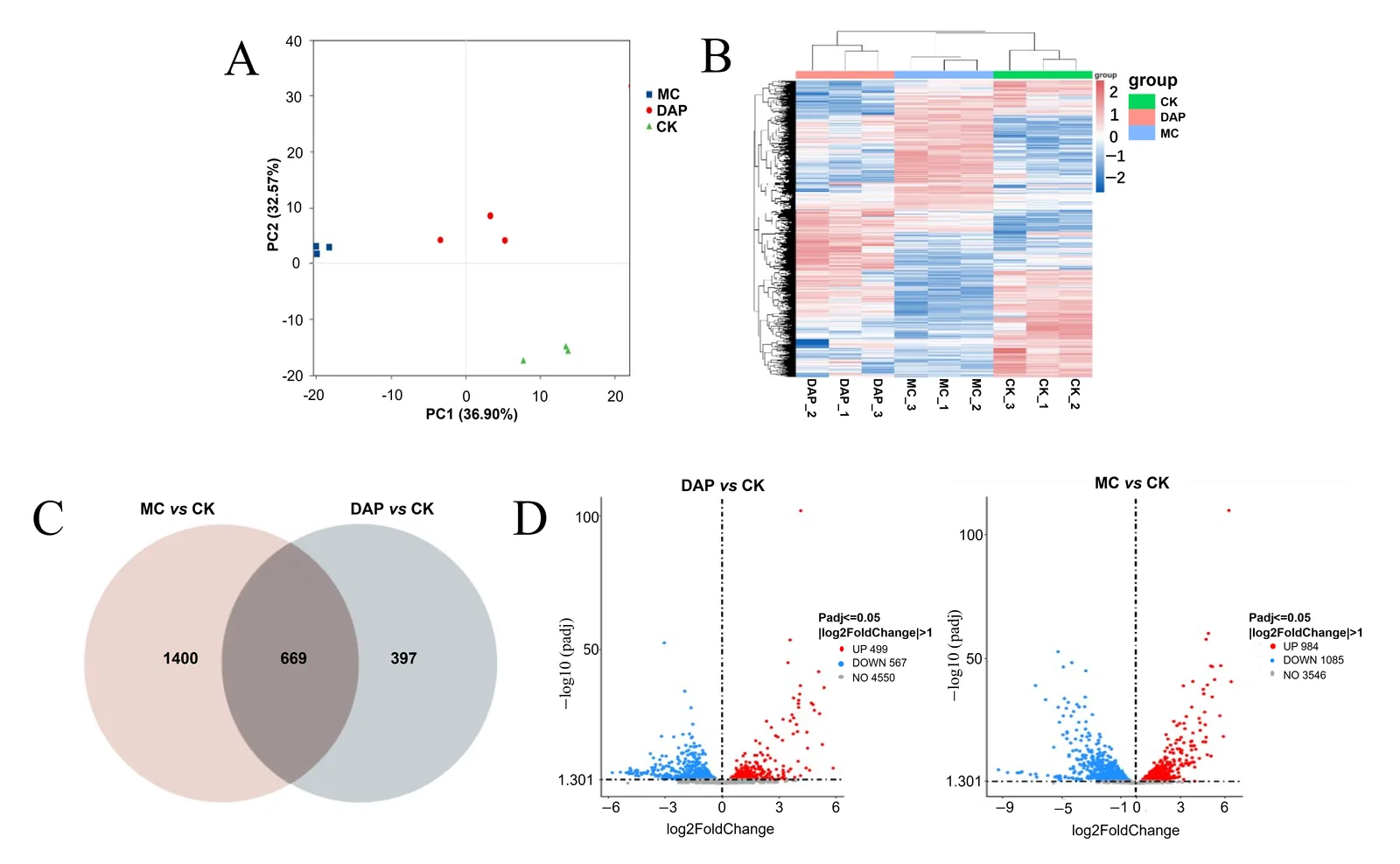
*R. pseudosolanacearum* transcriptome signatures influenced by coumarin derivatives. (**A**) Principal component analysis of transcriptome level supplemented with DAP and MC, (**B**) Hierarchical clustering heatmap of differentially expressed genes (DEGs), (**C**) Venn diagram illustrating the unique and shared DEGs, (**D**) Volcano plots displaying the distribution of significantly up-regulated and down-regulated DEGs.

To investigate the molecular mechanisms underlying the inhibition of *R. pseudosolanacearum* by the two coumarin derivatives, RNA-seq was performed. After filtering out low-quality reads, a total of 11.06 Gb of clean data was generated, averaging 1.23 Gb of high-quality data per sample, with all samples maintaining a Q30 quality score of at least 95.89% (Table S5). Principal component and hierarchical clustering analyses revealed that both coumarin treatments profoundly altered the transcriptomic profile of *R. pseudosolanacearum* compared to the control group, yet DAP and MC elicited distinct regulatory responses (Figure 6A,B). Specifically, DAP treatment induced the differential expression of 1066 genes (499 up-regulated and 567 down-regulated), whereas MC treatment exerted a more widespread impact, modulating 2069 DEGs (984 up-regulated and 1085 down-regulated) (Figure 6D). Among these, 669 DEGs were shared between the two treatment groups (Figure 6C). The substantially larger DEG count under MC treatment suggests a broader and more multifaceted mode of action against the pathogen compared with DAP. Furthermore, in both treatment groups, downregulated genes outnumbered upregulated ones, implying that transcriptional repression constitutes a dominant regulatory strategy during cellular stress adaptation.

#### 3.5.1. GO Enrichment Analysis of Differentially Expressed Genes

**Figure 7:**
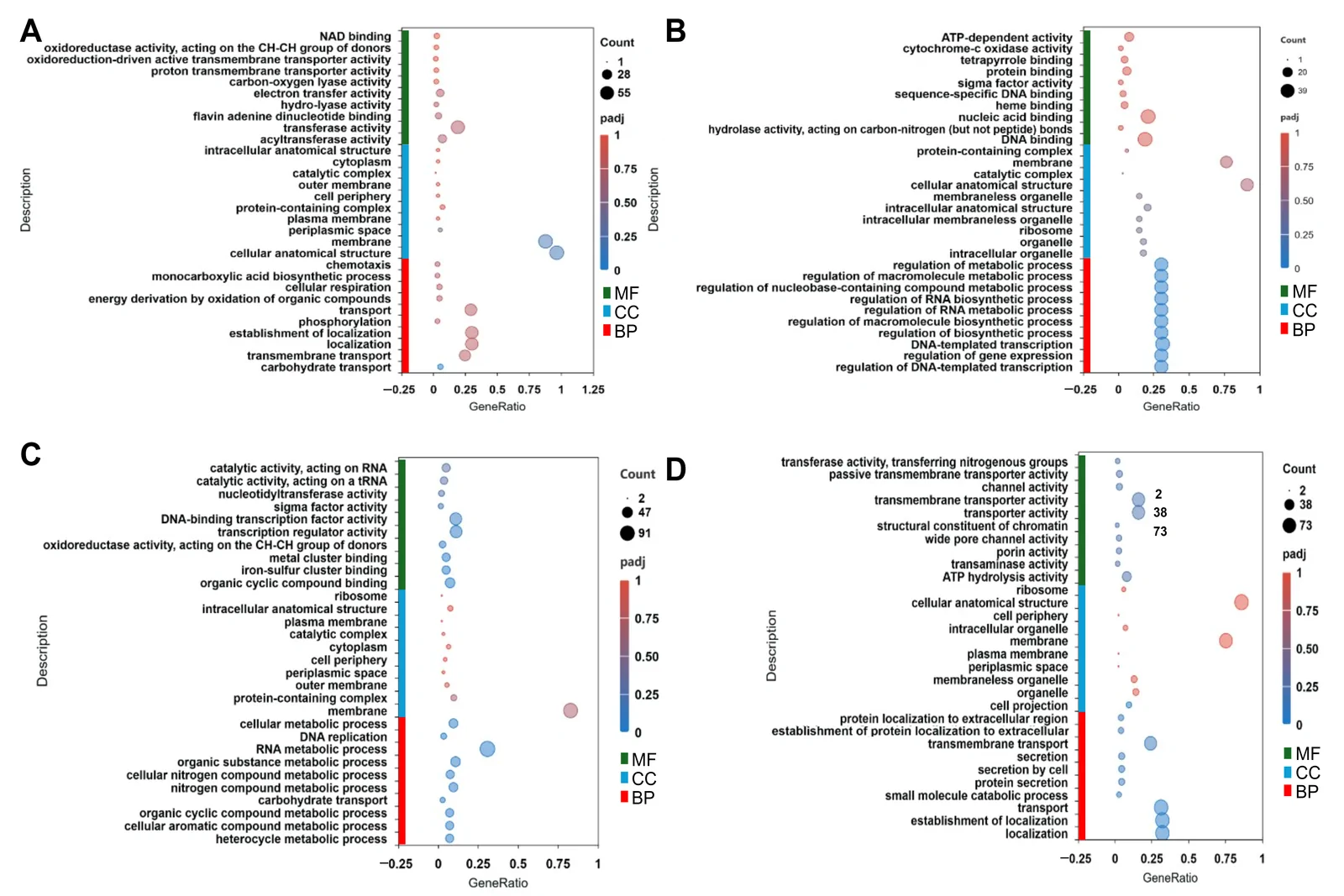
GO enrichment analysis of DEGs identified under DAP and MC treatments relative to the control. (**A**) Upregulated DEGs, DAP vs. CK. (**B**) Downregulated DEGs, DAP vs. CK. (**C**) Upregulated DEGs, MC vs. CK. (**D**) Downregulated DEGs, MC vs. CK. BP, biological process; CC, cellular component; MF, molecular function. The x-axis indicates the Gene Ratio (the proportion of DEGs annotated to a specific pathway relative to total DEGs). The size of each dot corresponds to the number of DEGs (Gene Count). The color scale represents the Benjamini–Hochberg adjusted *p*-value (*p* adj), ranging from blue (most statistically significant, *p* adj < 0.05) to red (less significant).

GO enrichment analysis categorized the DEGs into three main domains: biological process, cellular component, and molecular function. The results revealed distinct transcriptional regulatory and physiological survival strategies adopted by *R. pseudosolanacearum* under DAP versus MC treatments. In the DAP-treated group, upregulated DEGs were predominantly associated with carbohydrate transport, cellular anatomical structure, and membrane components (Figure 7A). Conversely, downregulated DEGs were significantly enriched in regulation of DNA-templated transcription, gene expression, biosynthesis/metabolism of RNA, macromolecules, and nucleobase-containing compounds (Figure 7B). These findings indicate that DAP imposes severe survival stress and metabolic suppression; it forces the pathogen to upregulate carbohydrate transport, while simultaneously stifling core regulatory pathways from DNA transcription to macromolecule synthesis, ultimately driving the cells into a state of transcriptional and growth arrest. In the MC-treated group, upregulated DEGs were primarily involved in DNA replication and a broad spectrum of metabolic pathways, including those of carbohydrates, nitrogen compounds, heterocycles, and organic cyclic/aromatic substances (Figure 7C). On the other hand, downregulated DEGs were predominantly enriched in molecular functions related to the binding of organic cyclic compounds, iron-sulfur clusters, and metal clusters (Figure 7D). This suggests that MC hyperactivates key carbon, nitrogen, and aromatic metabolic pathways, while suppressing the binding of organic cyclic compounds and iron-sulfur/metal clusters, thereby impeding respiratory electron transport chain efficiency.

#### 3.5.2. KEGG Enrichment Analysis of Differentially Expressed Genes

**Figure 8:**
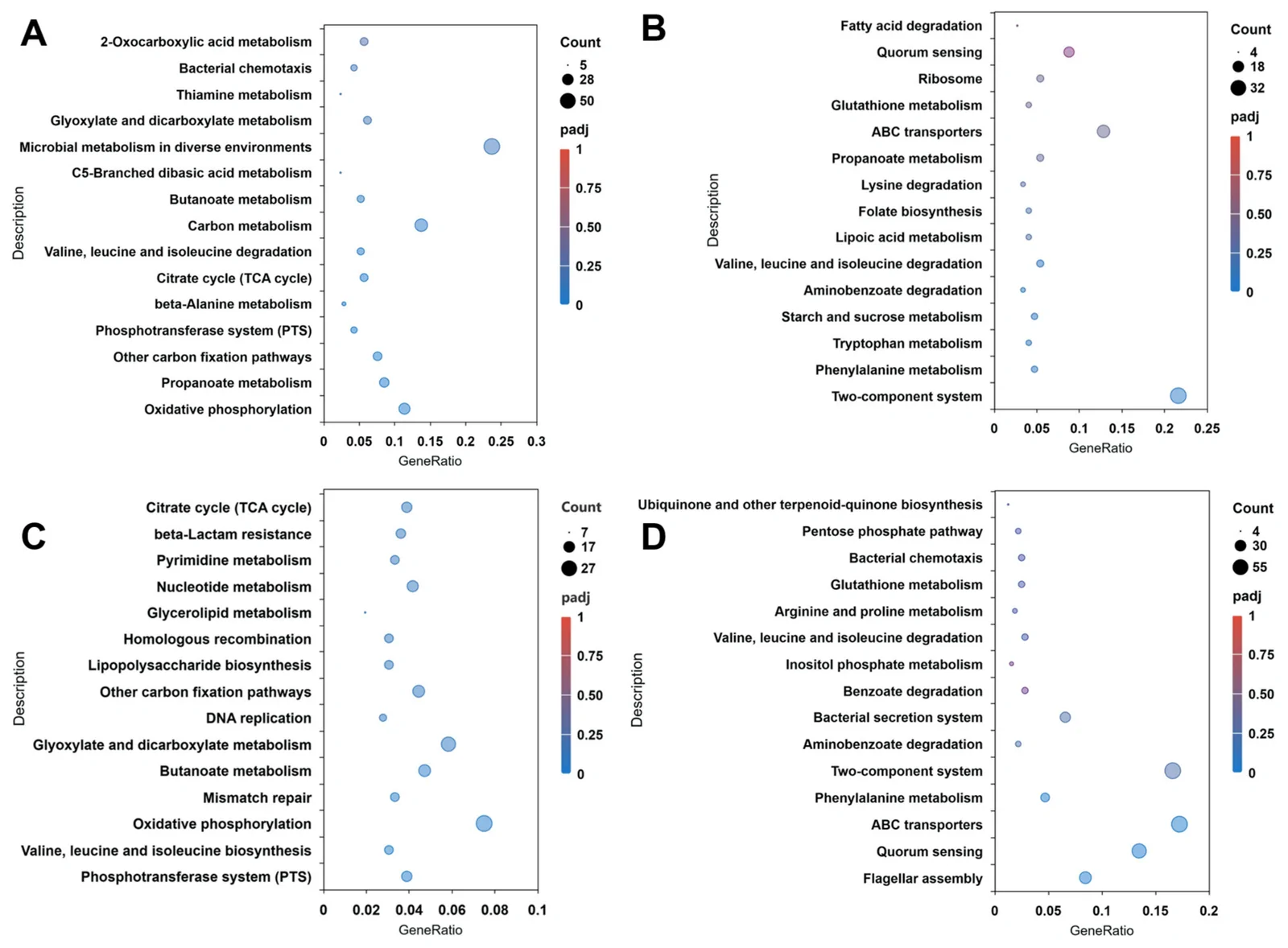
KEGG enrichment analysis of DEGs identified under DAP and MC treatments. (**A**) Upregulated DEGs, DAP vs. CK. (**B**) Downregulated DEGs, DAP vs. CK. (**C**) Upregulated DEGs, MC vs. CK. (**D**) Downregulated DEGs, MC vs. CK. The x-axis indicates the Gene Ratio (the proportion of DEGs annotated to a specific pathway relative to total DEGs). The size of each dot corresponds to the number of DEGs. The color scale represents the Benjamini–Hochberg adjusted *p*-value (*p* adj), ranging from blue (most statistically significant, *p* adj < 0.05) to red (less significant).

KEGG enrichment analysis was performed for cells following DAP and MC treatments. For DAP treatment, upregulated DEGs were primarily enriched in pathways such as oxidative phosphorylation, propanoate metabolism, other carbon fixation pathways, phosphotransferase system, citrate cycle, beta-alanine metabolism, and carbon metabolism (Figure 8A). Conversely, downregulated DEGs were enriched in two-component systems, aminobenzoate degradation, lipoic acid metabolism, folate biosynthesis, phenylalanine, tryptophan, and starch and sucrose metabolism (Figure 8B).

For MC treatment, upregulated DEGs were enriched in DNA replication, mismatch repair, oxidative phosphorylation, and alternative amino acid networks (Figure 8C). Meanwhile, downregulated DEGs were enriched in two-component systems, ABC transporters, quorum sensing, flagellar assembly, and bacterial secretion systems (Figure 8D).

**Figure 9:**
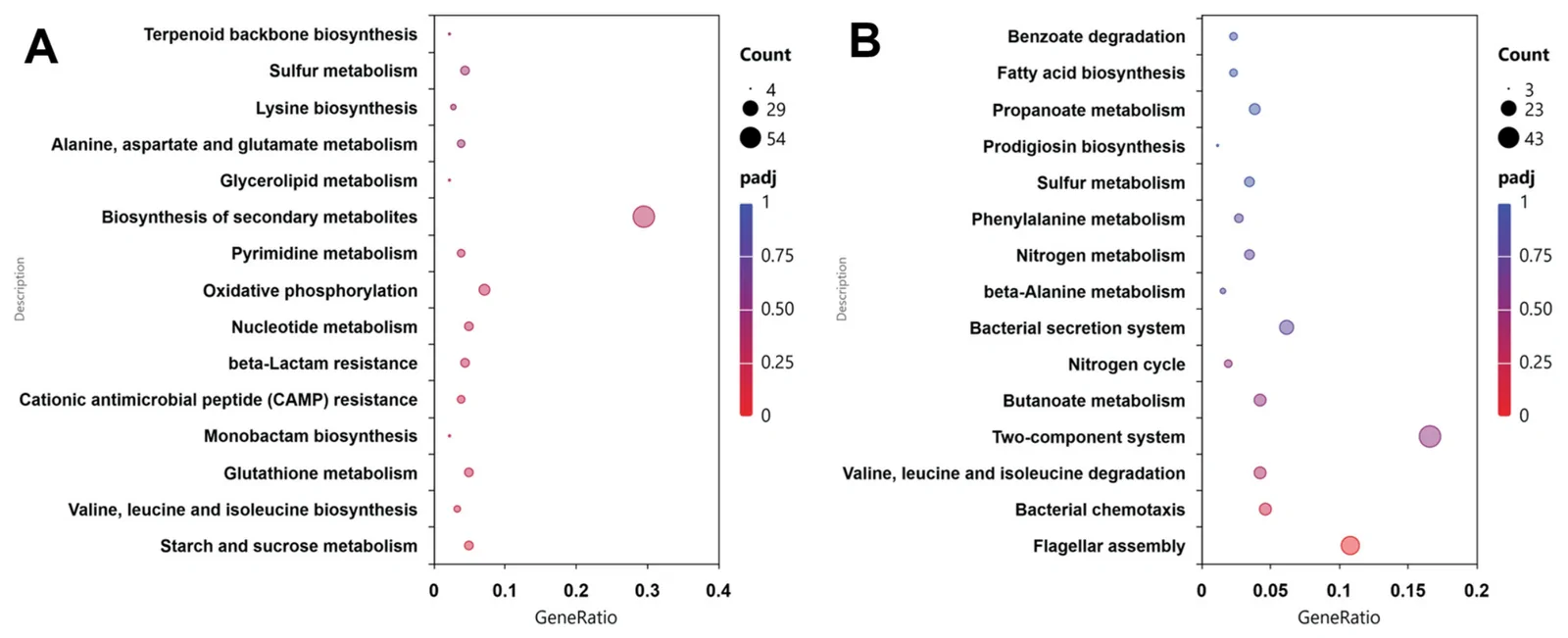
KEGG pathway enrichment analysis of DEGs between MC and DAP treatments. (**A**) Upregulated DEGs, MC vs. DAP. (**B**) Downregulated DEGs, MC vs. DAP. The size of each dot corresponds to the number of DEGs. The color scale represents the Benjamini–Hochberg adjusted *p*-value (*p* adj), ranging from red (most statistically significant, *p* adj < 0.05) to blue (less significant).

Furthermore, direct pairwise comparison between MC and DAP treatments revealed distinct enrichment patterns. For MC vs. DAP, upregulated DEGs were significantly enriched in biosynthesis of starch and sucrose metabolism, glutathione metabolism, valine, leucine and isoleucine biosynthesis, monobactam biosynthesis, oxidative phosphorylation, and antimicrobial resistance pathways (beta-lactam resistance and cationic antimicrobial peptide resistance) (Figure 9A). Conversely, downregulated DEGs were highly enriched in flagellar assembly, bacterial chemotaxis, two-component system, and valine, leucine and isoleucine degradation (Figure 9B).

Overall, the enrichment trends derived from the direct comparison (MC vs. DAP) were largely consistent with those observed in individual treatments relative to the CK. The direct comparison further demonstrated that MC exerted a substantially stronger suppressive effect on flagellar assembly, bacterial chemotaxis and two-component system than DAP, whereas DAP showed a more pronounced impact on amino acid and carbohydrate metabolism and biosynthesis of other secondary metabolites.

#### 3.5.3. Hierarchical Clustering Analysis of Core Pathway-Associated DEGs

**Figure 10:**
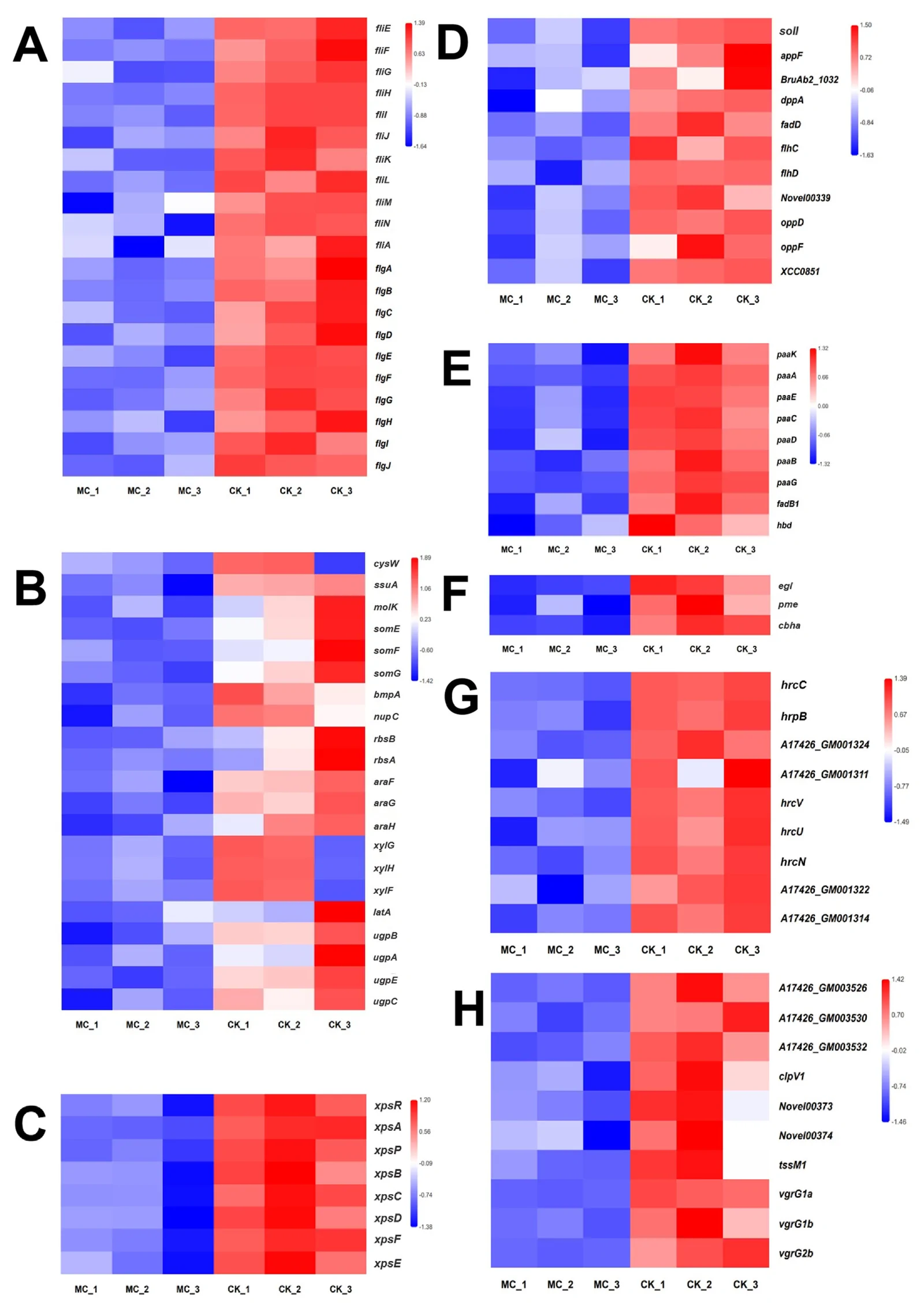
Hierarchical clustering heatmap of key DEGs in *R. pseudosolanacearum* in response to MC treatment. Heatmaps show the relative expression levels of DEGs in three biological replicates of DAP-treated (DAP_1, DAP_2, DAP_3) and control groups (CK_1, CK_2, CK_3). (**A**) Flagellar assembly, (**B**) Mineral and organic ion transporter, (**C**) EPS biosynthesis, (**D**) Quorum sensing, (**E**) Phenylalanine degradation, (**F**) Plant cell wall-degrading enzymes, (**G**) T3SS, (**H**) T6SS. The color bar represents the row Z-score derived from log_2_(*FPKM* + 1) values, where red indicates higher relative expression and blue indicates lower relative expression compared to the CK. Hierarchical clustering was performed using Euclidean distance with the complete linkage method.

Hierarchical clustering analysis was performed on key DEGs to examine the expression patterns of functional nodes following MC treatment (Figure 10), and full gene list is provided in Table S6. Regarding motility and communication, MC treatment induced a significant down-regulation of a large gene cluster encoding the bacterial flagellar motor structure and its associated secretion apparatus (e.g., *fliE*/*F*, *flgA*, and *flhA*; Figure 10A). Concurrently, genes involved in the quorum sensing pathway (*solI*) and the oligopeptide transport system (e.g., *oppD* and *dppA*) were markedly repressed (Figure 10D). Beyond signaling and motility, genes mediating nutrient acquisition were also affected. Functional components within the ABC transport system—including those for ribose system (e.g., *rbsA*, *rbsB*), arabinose system (e.g., *araF*, *araG*, *araH*), xylose system (e.g., *xylF*, *xylG* and *xylH*), glycerol-3-phosphate system (e.g., *ugpA* and *ugpB*), other oligosaccharide/monosaccharide transport components (e.g., *malK*, *msnX*, *somE*) and ion transport (e.g., *cysW* and *ssuA*)—showed significant downregulation (Figure 10B). Furthermore, key genes involved in the phenylalanine degradation pathway (e.g., *paaA* and *paaK*) exhibited clustered down-regulation (Figure 10E), underscoring that MC treatment severely compromises nutrient acquisition and specific amino acid catabolism in *R. pseudosolanacearum*.

Crucially, MC treatment reduced the transcript levels of key virulence-related gene clusters. Expression of the EPS biosynthetic operon (e.g., *epsA* and *epsB*) and its regulator *xpsR* (Figure 10C) were markedly reduced. Similarly, genes encoding critical plant cell wall-degrading enzymes, including endoglucanase egl, pectinesterase pme, and cellobiohydrolase cbhA, were uniformly downregulated (Figure 10F). Most notably, core structural and effector components of macromolecular secretion systems—including the T3SS apparatus components (e.g., *hrpB3* and *hrcV*; Figure 10G) and the Type VI secretion system (T6SS, e.g., *clpV1* and *tssM1*; Figure 10H)—were heavily suppressed. Together, these transcriptomic patterns reveal a comprehensive repression of gene networks associated with motility, nutrient transport, and pathogenicity in *R. pseudosolanacearum*.

**Figure 11:**
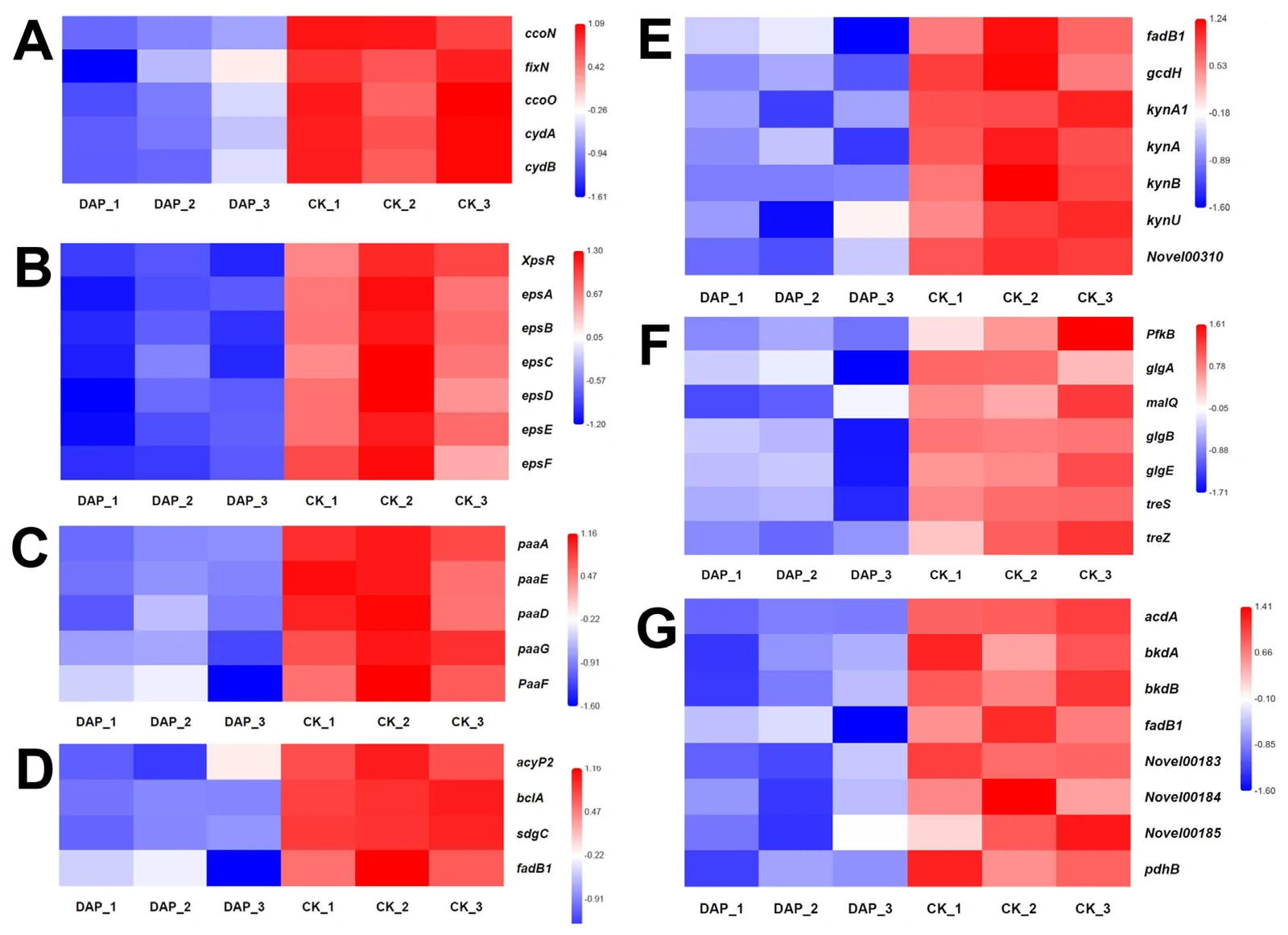
Hierarchical clustering heatmap of key DEGs in *R. pseudosolanacearum* in response to DAP treatment. Heatmaps show the relative expression levels of DEGs in three biological replicates of DAP-treated (DAP_1, DAP_2, DAP_3) and control groups (CK_1, CK_2, CK_3). (**A**) aerobic respiration, (**B**) EPS biosynthesis, (**C**) Phenylalanine degradation pathway, (**D**) Aminobenzoate degradation, (**E**) Tryptophan metabolism, (**F**) Starch and sucrose metabolism, (**G**) Valine, leucine, and isoleucine degradation. The color bar represents the row Z-score derived from log_2_(*FPKM* + 1) values, where red indicates higher relative expression and blue indicates lower relative expression compared to the CK. Hierarchical clustering was performed using Euclidean distance with the complete linkage method.

In contrast to the multi-layered defenses targeted by MC, DAP treatment elicited a distinct and multi-targeted suppressive effect on the respiratory machinery, carbon networks, peripheral amino acid catabolism, and primary virulence factor, with the full gene list provided in Table S7. Specifically, DAP exposure disrupted the electron transport chain of *R. pseudosolanacearum*, as evidenced by the concomitant downregulation of genes encoding key components of the aerobic respiratory system, including the -type cytochrome c oxidase subunits ccoN and ccoO, alongside the alternative terminal oxidase subunits cydA and cydB (Figure 11A). This respiratory blockade coincided with a significant downregulation of central and storage carbon pathways within starch and sucrose metabolism, where the 6-phosphofructokinase gene *pfkB*, the core glycogen biosynthesis genes *glgA, glgB* and *malQ,* and the trehalose synthesis-related genes *treS* and *treZ* were drastically suppressed (Figure 11F). DAP extensively bottlenecked the utilization of alternative carbon and nitrogen sources. Within the valine, leucine, and isoleucine degradation pathways, genes encoding the branched-chain -ketoacid dehydrogenase complex components bkdA, bkdB, and pdhB, the acyl-CoA dehydrogenase gene *acdA* (Figure 11G), and the bifunctional 3-hydroxyacyl-CoA dehydrogenase gene *fadB1* were uniformly downregulated (Figure 11D). This metabolic arrest extended further to peripheral amino acid catabolism and aromatic degradation networks. In the phenylalanine degradation pathway, the expression of the *paaD, paaE, paaG* gene cluster was robustly suppressed (Figure 11C). Similarly, the tryptophan metabolism pathway involving *gcdH*, *kynA*, *kynA1*, *kynB*, and *kynU* (Figure 11E), as well as the aminobenzoate degradation pathway featuring *acyP2*, *bclA*, *sdgC*, and the intersecting *fadB1* (Figure 11D), were significantly downregulated. Collectively, these perturbations indicate a severe impairment of metabolic flexibility and cellular detoxification capacities under DAP stress. Crucially, beyond energetic and metabolic strangulation, DAP treatment directly dismantled the primary virulence apparatus of *R. pseudosolanacearum*. This was accomplished by robustly downregulating the master virulence regulator *xpsR*, alongside the core EPS biosynthetic operon genes, including *epsABCDEFP* (Figure 11B).

## 4. Discussion

The global transcriptomic profiles aligned well with the observed phenotypic changes, where the downregulation of key gene clusters supported the reductions in bacterial motility, EPS production, and biofilm formation in vitro. In the microdilution assays, DAP demonstrated superior antibacterial efficacy against *R. pseudosolanacearum* compared to MC, characterized by a lower MIC 62.5 μg/mL vs. 125 μg/mL and MBC 250 μg/mL vs. 1000 μg/mL. Interestingly, transcriptomic profiling revealed both shared and distinct response patterns between the two coumarin derivatives. On one hand, the two compounds exhibited notable divergence in the magnitude of response: MC induced a substantially broader perturbation with 2069 DEGs, compared to 1066 DEGs regulated by DAP, indicating that MC elicits a more extensive reprogramming of the bacterial transcriptional network. On the other hand, a core set of 669 DEGs was co-regulated by both treatments. This shared transcriptomic response directly aligns with their common impact on key pathways, notably upregulating butanoate metabolism and the TCA cycle while downregulating the two-component system and ABC transporters.

The phenotypic data revealed that MC was a more potent inhibitor of *R. pseudosolanacearum* swimming motility and biofilm formation than DAP, inducing an early reduction in swimming diameter at 20 μg/mL and near-complete eradication of biofilms at 160 μg/mL. This phenotypic disruption is robustly supported by the transcriptomic data. MC exposure significantly downregulated a series of core gene clusters governing flagellar assembly (*fliE-N*, *flhA*, and *flgA-J*), thereby impairing the bacterial motility apparatus. Indeed, the direct comparative analysis confirmed that flagellar assembly and bacterial chemotaxis were significantly down-regulated in MC compared to DAP, directly explaining the superior motility inhibition observed for MC. Regarding intercellular communication, MC significantly suppressed the expression of *solI*, which encodes an N-acyl-homoserine lactone synthase and serves as a key component of the SolI/R QS system. This system regulates the *lecM*, *aidA*, and *aidC* loci, all of which are closely associated with bacterial virulence [41]. Furthermore, genes encoding components of the oligopeptide transport system (*appF*, *oppD/F*, and *dppA*), which mediates cell-to-cell interactions, also exhibited a marked downward trend. This multi-layered disruption of signaling and collective behavior is highly concordant with the classic quorum quenching mechanism [42], which effectively severs the bacterial signal flow before the pathogen can coordinate a synchronized attack. Notably, MC targeted the T3SS—a critical virulence factor of the pathogen—by significantly suppressing the expression of its core structural genes (*hrcV*, *hrcU*, and *hrcC*), the ATPase-encoding gene (*hrcN*, which provides the necessary bioenergetic driving force), as well as key regulatory genes (such as *hrpB*) [43]. This suppression potentially impairing the translocation of type III effectors into the host cytoplasm and attenuating the capacity to subvert host immune responses [44]. Simultaneously, the T6SS, which is involved in pathogenesis, interbacterial interaction, and competition [45], was also inhibited by MC, as evidenced by the significantly decreased expression of its core component genes *clpV1*, *tssM1*, and *vgrG*. Previous studies have established that *vgrG3* mutants exhibit significantly attenuated virulence and markedly alleviated wilting symptoms in host plants [46]. In conclusion, MC achieves a network-level blockade of the core pathogenicity by comprehensively suppressing quorum sensing, flagellar motility, and critical virulence secretion systems (T3SS and T6SS).

The mechanisms of action of DAP and MC exhibit substantial divergence. DAP displays potent bactericidal activity and a low MIC value, primarily attributable to its targeted disruption of central carbon metabolism and coenzyme biosynthesis pathways in *R. pseudosolanacearum*. This was further supported by the direct pairwise comparison (MC vs. DAP), where DAP demonstrated a significantly stronger impact on the carbohydrate metabolism and amino acid metabolism compared to MC. Previous studies on *R*. *solanacearum*—a phytopathogen of solanaceous crops—have demonstrated that DAP downregulates the expression of genes associated with fatty acid biosynthesis, lipopolysaccharide assembly, ribosomal biogenesis, and oxidative phosphorylation, thereby compromising cellular integrity and impairing energy metabolism [30,47]. In contrast, transcriptomic profiling of *R. pseudosolanacearum* revealed that DAP-mediated inhibition predominantly involves key pathways such as the two components system, phenylalanine metabolism, tryptophan metabolism, and starch and sucrose metabolism. Given the phylogenetic and physiological heterogeneity between the two strains, these differential transcriptional responses reflect lineage-specific adaptive strategies to DAP. Transcriptomic analysis of *R. pseudosolanacearum* revealed that DAP specifically represses the *ccoO*/*ccoN* genes—core components of the cytochrome *cbb_3_* oxidase operon. The *cbb_3_*-type cytochrome c oxidase is a high-affinity terminal oxidase involved in microaerobic respiration [48] and is required for virulence in host plant [49], suggesting that DAP may compromise the energy metabolism required for bacterial adaptation within plant tissues. Concurrently, DAP downregulates multiple carbon/nitrogen energy supplying pathways, including phenylacetate catabolism, tryptophan degradation, and starch/sucrose utilization. Notably, within the tryptophan degradation pathway, the enzymes KynA1, KynB, and KynU catalyze the conversion of kynurenine to anthranilate, with concomitant release of L-alanine. Anthranilate serves not only as a key precursor for quinolone signal molecule biosynthesis in bacteria, but can also be further degraded to catechol, which ultimately feeds into the energy metabolic cycle [50]. Hamilton et al. demonstrated that disruption of trehalose catabolism led to significantly reduced xylem colonization, competitive fitness, and virulence [51]. Although the exact sugar composition of eucalyptus xylem sap remains uncharacterized, these findings highlight trehalose as an important metabolic resource for the pathogen. Notably, DAP treatment down-regulated genes encoding trehalose synthase (treS) and malto-oligosyltrehalose trehalohydrolase (treZ), suggesting that DAP may impair the bacterial capacity to synthesize and maintain the metabolic homeostasis of this essential nutrient. Additionally, glycogen serves as another critical carbon and energy reserve; repression of its biosynthesis related genes (*glgA*/*B/E*) further constrains the pathogen’s energy supply. Meanwhile, phosphofructokinase (PfkB), a component of the *ptsP*-*pfkB* regulon, plays a key role in carbohydrate transport by phosphorylating incoming sugar substrates concomitant with their translocation across the cell membrane [52]. The DAP-induced down-regulation of *pfkB* may impair carbohydrate translocation. Coordinated repression of these genes impairs carbon source utilization, depletes energy reserves, and compromises the microaerobic respiration chain—collectively eroding metabolic flexibility and stress tolerance.

MC and DAP exhibit distinctly bifurcated antimicrobial mechanisms against *R*. *pseudosolanacearum*. MC acts primarily repressing flagellar motility and secretion machinery, whereas DAP acts predominantly as a metabolic disruptor that starves the bacterium of energy and key nutrients. Intriguingly, both compounds shared a common downstream target by downregulating the master virulence regulator *xpsR* and its downstream eps biosynthetic operon, thereby potently impeding EPS accumulation. Given that EPS-mediated vascular occlusion is a critical contributor to host wilting, the in vitro suppression of EPS-related traits suggests that both agents may attenuate bacterial wilt development, warranting further in planta evaluation.

Conventional chemical control strategies, particularly when applied intensively with a single target site, may pose a high risk of accelerating the development of pathogen resistance and adversely affect soil ecological equilibrium. In contrast, MC and DAP operate via two distinct yet partially convergent mechanisms of action, offering a multi-target framework to mitigate resistance risks. Although their in planta efficacy requires validation through *Eucalyptus* inoculation and field trials, and their potential synergy remains to be evaluated, this study provides a theoretical foundation for developing multi-target botanical antibacterial formulations for sustainable Eucalyptus bacterial wilt disease management in China.

## 5. Conclusions

In summary, this study provides an in vitro phenotypic and transcriptomic evaluation of the antimicrobial and anti-virulence activities of DAP and MC against the eucalyptus-derived *R. pseudosolanacearum* strain evaluated here. The phenotypic combination transcriptome characteristics reveal distinct impacts between these two chemical scaffolds. DAP exhibits higher bactericidal potency, associated with the down-regulation of genes governing peripheral carbon and nitrogen metabolic networks and aerobic respiratory chains. Conversely, at sub-lethal concentrations, MC specifically suppresses functions critical to active pathogenesis, including flagellar assembly, quorum sensing, and the T3SS and T6SS, thereby impairing bacterial motility, and effector translocation components. Direct pairwise comparison further confirmed this functional specialization, demonstrating that MC exerts a significantly stronger suppressive effect on motility and virulence secretion systems than DAP, whereas DAP more profoundly impairs energy and nutrient metabolism pathways. Notably, both compounds converge downstream to decrease the transcription of the master regulator *xpsR* and the *epsA-P* biosynthetic operon, consistently reducing extracellular polysaccharide accumulation. Collectively, these regulatory profiles provide a clear molecular rationale for subsequent evaluation of these botanical compounds in eucalyptus bacterial wilt models.

## Data Availability

The transcriptome of *R. pseudosolanacearum* have been deposited at SRA database with the accession number PRJNA1484465.

## Supplementary Materials

The following supporting information can be downloaded at: https://www.mdpi.com/article/10.3390/microorganisms14081799/s1, Table S1: Effect of DAP and MC at different concentrations on the cell viability of *R. pseudosolanacearum*; Table S2: Joint comparison of DAP and MC treatments on the swimming motility diameter of *R. pseudosolanacearum* across equivalent concentrations after 48 h of incubation; Table S3: Joint comparison of DAP and MC treatments on the biofilm formation of *R. pseudosolanacearum* across equivalent concentrations after 36 h of incubation; Table S4: Joint comparison of DAP and MC treatments on the EPS production of *R. pseudosolanacearum* across equivalent concentrations after 24 h of incubation; Table S5: Sequencing and quality control statistics of the *R. pseudosolanacearum* transcriptome; Table S6: Differential expression of genes in core metabolic pathways of the *R. pseudosolanacearum* under MC treatment; Table S7: Differential expression of genes in core metabolic pathways of the *R. pseudosolanacearum* under DAP treatment.

## Abbreviations

The following abbreviations are used in this manuscript:

DAP	daphnetin
MC	6-methylcoumarin
EPS	extracellular polysaccharides
RSSC	*Ralstonia solanacearum* species complex
LPS	lipopolysaccharide
T3SS	Type III secretion system
T6SS	Type VI secretion system
DEGs	differentially expressed genes
DMSO	dimethyl sulfoxide
PBS	phosphate-buffered saline
GO	Gene Ontology
KEGG	Kyoto Encyclopedia of Genes and Genomes

## References

[1] Chachar Z., Xue X., Fang J., Chen M., Jiarui C., Chen W., Ahmed N., Chachar S., Narejo M.-N., Ahmed N. (2025). Key mechanisms of plant-*Ralstonia solanacearum* interaction in bacterial wilt pathogenesis. Front. Microbiol..

[2] Yao J., Allen C. (2006). Chemotaxis is required for virulence and competitive fitness of the bacterial wilt pathogen *Ralstonia solanacearum*. J. Bacteriol..

[3] Corral J., Sebastià P., Coll N.S., Barbé J., Aranda J., Valls M. (2020). Twitching and swimming motility play a role in *Ralstonia solanacearum* pathogenicity. mSphere.

[4] Fegan M., Prior P., Allen C., Prior P., Hayward A.C. (2005). How complex is the “*Ralstonia solanacearum* species complex”?. Bacterial Wilt: The Disease and the Ralstonia solanacearum Species Complex.

[5] García R.O., Kerns J.P., Thiessen L. (2019). *Ralstonia solanacearum* species complex: A quick diagnostic guide. Plant Health Prog..

[6] Safni I., Cleenwerck I., De Vos P., Fegan M., Sly L., Kappler U. (2014). Polyphasic taxonomic revision of the *Ralstonia solanacearum* species complex: Proposal to emend the descriptions of *Ralstonia solanacearum* and *Ralstonia syzygii* and reclassify current *R. syzygii* strains as *Ralstonia syzygii* subsp. syzygii subsp. nov., *R. solanacearum* phylotype IV strains as *Ralstonia syzygii* subsp. indonesiensis subsp. nov., banana blood disease bacterium strains as *Ralstonia syzygii* subsp. celebesensis subsp. nov. and *R. solanacearum* phylotype I and III strains as *Ralstonia pseudosolanacearum* sp. nov. Int. J. Syst. Evol. Microbiol..

[7] Carstensen G.D., Venter S.N., Wingfield M.J., Coutinho T.A. (2017). Two Ralstonia species associated with bacterial wilt of *Eucalyptus*. Plant Pathol..

[8] Xu W., Li Y., Jiang N., Li H., Xue H. (2025). Genomic and transcriptomic characterization of the eucalyptus bacterial wilt pathogen. Acta Phytopathol. Sin..

[9] Fujiwara A., Fujisawa M., Hamasaki R., Kawasaki T., Fujie M., Yamada T. (2011). Biocontrol of *Ralstonia solanacearum* by treatment with lytic bacteriophages. Appl. Environ. Microbiol..

[10] Wang Z., Luo W., Cheng S., Zhang H., Zong J., Zhang Z. (2023). *Ralstonia solanacearum*-a soil borne hidden enemy of plants: Research development in management strategies, their action mechanism and challenges. Front. Plant Sci..

[11] Lin T., Lu Y., Zeng W., Lin X., Lai R., Tang M., Peng S. (2020). Toxicity effects of common fungicides on tobacco bacterial wilt caused by *Ralstonia solanacearum*. J. Agric..

[12] Han Y.K., Han K.S., Lee S.C., Kim S. (2011). Control of bacterial wilt of tomato using copper hydroxide. Korean J. Pestic. Sci..

[13] Sang S., Li S., Fan W., Wang N., Gao M., Wang Z. (2019). Zinc thiazole enhances defense enzyme activities and increases pathogen resistance to *Ralstonia solanacearum* in peanut (*Arachis hypogaea*) under salt stress. PLoS ONE.

[14] Mao L., Jiang H., Wang Q., Yan D., Cao A. (2017). Efficacy of soil fumigation with dazomet for controlling ginger bacterial wilt (*Ralstonia solanacearum*) in China. Crop Prot..

[15] Ritchie D.F., Dittapongpitch V. (1991). Copper- and streptomycin-resistant strains and host differentiated races of *Xanthomonas campestris* pv. vesicatoria in North Carolina. Plant Dis..

[16] Dasgupta S., Meisner C.M., Wheeler D., Lam N.T., Xuyen K. (2005). Pesticide Poisoning of Farm Workers: Implications of Blood Test Results from Vietnam.

[17] Pham M.H., Sebesvari Z., Tu B.M., Pham H.V., Renaud F.G. (2011). Pesticide pollution in agricultural areas of Northern Vietnam: Case study in Hoang Liet and Minh Dai communes. Environ. Pollut..

[18] Yuliar, Nion Y.A., Toyota K. (2015). Recent trends in control methods for bacterial wilt diseases caused by *Ralstonia solanacearum*. Microbes Environ..

[19] Du T., Zhu W., Zhang C., Liang X., Shu Y., Zhou J., Zhang M., He Y., Tu J., Feng Y. (2025). Bacteriostatic activity and resistance mechanism of *Artemisia annua* extract against *Ralstonia solanacearum* in pepper. Plants.

[20] Mendoza-Buenrostro E., Rangel-Vargas E., Gómez-Aldapa C.A., Velázquez-Jiménez R., Torres-Vitela M.R., Castro-Rosas J. (2025). Effective use of microbial and plant-based alternatives in tomato pathogen control: A comprehensive review. Plant Pathol..

[21] Yang L., Wei Z., Li S., Xiao R., Xu Q., Ran Y., Ding W. (2021). Plant secondary metabolite, daphnetin reduces extracellular polysaccharides production and virulence factors of *Ralstonia solanacearum*. Pestic. Biochem. Physiol..

[22] Do H.T.T., Nguyen T.H., Nghiem T.D., Nguyen H.T., Choi G.J., Ho C.T., Dang Q.L. (2021). Phytochemical constituents and extracts of the roots of *Scutellaria baicalensis* exhibit in vitro and in vivo control efficacy against various phytopathogenic microorganisms. S. Afr. J. Bot..

[23] Mohamed A.A., Behiry S.I., Younes H.A., Ashmawy N.A., Salem M.Z.M., Márquez-Molina O., Barbabosa-Pilego A. (2019). Antibacterial activity of three essential oils and some monoterpenes against *Ralstonia solanacearum* phylotype II isolated from potato. Microb. Pathog..

[24] Niu S., Liu D., Proksch P., Shao Z., Lin W. (2015). New polyphenols from a deep sea *Spiromastix sp.* fungus, and their antibacterial activities. Mar. Drugs.

[25] Paret M.L., Cabos R., Kratky B.A., Alvarez A.M. (2010). Effect of plant essential oils on *Ralstonia solanacearum* race 4 and bacterial wilt of edible ginger. Plant Dis..

[26] Robe K., Izquierdo E., Vignols F., Rouached H., Dubos C. (2021). The coumarins: Secondary metabolites playing a primary role in plant nutrition and health. Trends Plant Sci..

[27] Rastija V., Vrandečić K., Ćosić J., Kanižai Šarić G., Majić I., Karnaš M. (2023). Prospects of computer-aided molecular design of coumarins as ecotoxicologically safe plant protection agents. Appl. Sci..

[28] Ma H., Wang K., Wang B., Wang Z., Liu Y., Wang Q. (2024). Design, synthesis, and biological activities of novel coumarin derivatives as pesticide candidates. J. Agric. Food Chem..

[29] Chen J., Yu Y., Li S., Ding W. (2016). Resveratrol and coumarin: Novel agricultural antibacterial agent against *Ralstonia solanacearum* in vitro and in vivo. Molecules.

[30] Yang L., Guan D., Valls M., Ding W. (2021). Sustainable natural bioresources in crop protection: Antimicrobial hydroxycoumarins induce membrane depolarization-associated changes in the transcriptome of *Ralstonia solanacearum*. Pest Manag. Sci..

[31] Yang L., Wang Y., He X., Xiao Q., Han S., Jia Z., Li S., Ding W. (2021). Discovery of a novel plant-derived agent against *Ralstonia solanacearum* by targeting the bacterial division protein FtsZ. Pestic. Biochem. Physiol..

[32] Han S., Yang L., Wang Y., Ran Y., Li S., Ding W. (2021). Preliminary studies on the antibacterial mechanism of a new plant-derived compound, 7-methoxycoumarin, against *Ralstonia solanacearum*. Front. Microbiol..

[33] Yang L., Wu L., Yao X., Zhao S., Wang J., Li S., Ding W. (2018). Hydroxycoumarins: New, effective plant-derived compounds reduce *Ralstonia pseudosolanacearum* populations and control tobacco bacterial wilt. Microbiol. Res..

[34] Humphries R.M., Ambler J., Mitchell S.L., Castanheira M., Dingle T., Hindler J.A., Koeth L., Sei K., on behalf of the CLSI Methods Development and Standardization Working Group of the Subcommittee on Antimicrobial Susceptibility Testing (2018). CLSI Methods Development and Standardization Working Group best practices for evaluation of antimicrobial susceptibility tests. J. Clin. Microbiol..

[35] Clough S.J., Flavier A.B., Schell M.A., Denny T.P. (1997). Differential expression of virulence genes and motility in *Ralstonia (Pseudomonas) solanacearum* during exponential growth. Appl. Environ. Microbiol..

[36] Oliveira L.S.S., Sirait B.A., Saha M.A., Sipayung J., Maretha M.V., Tarigan M., Duran A. (2023). Integrated management of eucalyptus bacterial wilt in Sumatra, Indonesia. Trop. Plant Pathol..

[37] Yao J., Allen C. (2007). The plant pathogen *Ralstonia solanacearum* needs aerotaxis for normal biofilm formation and interactions with its tomato host. J. Bacteriol..

[38] Peyraud R., Cottret L., Marmiesse L., Gouzy J., Genin S. (2016). A resource allocation trade-off between virulence and proliferation drives metabolic versatility in the plant pathogen *Ralstonia solanacearum*. PLoS Pathog..

[39] Young M.D., Wakefield M.J., Smyth G.K., Oshlack A. (2010). Gene ontology analysis for RNA-seq: Accounting for selection bias. Genome Biol..

[40] Mao X., Cai T., Olyarchuk J.G., Wei L. (2005). Automated genome annotation and pathway identification using the KEGG Orthology (KO) as a controlled vocabulary. Bioinformatics.

[41] Li P., Bez C., Zhang Y., Deng Y., Venturi V. (2024). N-acyl homoserine lactone cell-cell diffusible signalling in the *Ralstonia solanacearum* species complex. Mol. Plant Pathol..

[42] García-Contreras R., Maeda T., Wood T.K. (2013). Resistance to quorum-quenching compounds. Appl. Environ. Microbiol..

[43] Chen M., Zhang W., Han L., Ru X., Cao Y., Hikichi Y., Ohnishi K., Pan G., Zhang Y. (2022). A CysB regulator positively regulates cysteine synthesis, expression of type III secretion system genes and pathogenicity in *Ralstonia solanacearum*. Mol. Plant Pathol..

[44] Landry D., González-Fuente M., Deslandes L., Peeters N. (2020). The large, diverse, and robust arsenal of *Ralstonia solanacearum* type III effectors and their *in planta* functions. Mol. Plant Pathol..

[45] Zhang L., Xu J., Xu J., Zhang H., He L., Feng J. (2014). TssB is essential for virulence and required for type VI secretion system in *Ralstonia solanacearum*. Microb. Pathog..

[46] Asolkar T., Ramesh R. (2020). The involvement of the type six secretion system (T6SS) in the virulence of *Ralstonia solanacearum* on brinjal. 3 Biotech.

[47] Jiang L., Zeng Z., Kaur A., Yang W., Huang H., Li S., Jiang S. (2025). Pathogenic *Ralstonia solanacearum* in agriculture: A review of prevention and control strategies based on natural products. Curr. Microbiol..

[48] Ailloud F., Lowe T.M., Robène I., Cruveiller S., Allen C., Prior P. (2016). In planta comparative transcriptomics of host-adapted strains of *Ralstonia solanacearum*. PeerJ.

[49] Colburn-Clifford J., Allen C. (2010). A cbb(3)-type cytochrome C oxidase contributes to *Ralstonia solanacearum* R3bv2 growth in microaerobic environments and to bacterial wilt disease development in tomato. Mol. Plant-Microbe Interact..

[50] Hwang H.J., Li X.H., Kim S.K., Lee J.H. (2022). Anthranilate acts as a signal to modulate biofilm formation, virulence, and antibiotic tolerance of *Pseudomonas aeruginosa* and surrounding bacteria. Microbiol. Spectr..

[51] Hamilton C.D., Steidl O.R., MacIntyre A.M., Hendrich C.G., Allen C. (2021). *Ralstonia solanacearum* depends on catabolism of myo-inositol, sucrose, and trehalose for virulence in an infection stage-dependent manner. Mol. Plant-Microbe Interact..

[52] Saleem T., Zou H., Zhuo T., Fan X. (2024). A LysR-type regulator influencing swimming motility, galactose utilization, and virulence in *Ralstonia solanacearum* GMI1000. Chem. Biol. Technol. Agric..

