# Supplementary material for: Comparative Phenotypic and Transcriptomic Analysis Reveals Distinct Yet Partially Convergent Mechanisms of Daphnetin and 6-Methylcoumarin Against Eucalyptus-Derived Ralstonia pseudosolanacearum
Source: Microorganisms. 2026 Aug 14;14(8):1799. doi: 10.3390/microorganisms14081799 (PMC13515791; doi:10.3390/microorganisms14081799)
Supplement: Supplementary file 1 [file microorganisms-14-01799-s001.zip › microorganisms-4450759-supplementary.pdf]

Table S1. Effect of DAP and MC at different concentrations on the cell viability of *R. pseudosolanacearum.*

Treatment	Concentration (μg /mL)	Mean CFU/mL
Control (CK)	0	$(1.25 \pm 0.12) \times 10^{8}$ a
Vehicle (DMSO)	0	$(1.21 \pm 0.10) \times 10^{8}$ a
DAP	31.25	$(8.50 \pm 0.42) \times 10^{6}$ b
	62.5	$(2.10 \pm 0.24) \times 10^{4}$ c
	125	$(1.22 \pm 0.18) \times 10^{3}$ d
	250-2000	ND
MC	31.25	$(6.23 \pm 0.48) \times 10^{7}$ a
	62.5	$(2.84 \pm 0.30) \times 10^{7}$ b
	125	$(3.55 \pm 0.38) \times 10^{5}$ c
	250	$(1.80 \pm 0.25) \times 10^{3}$ d
	500	$(1.50 \pm 0.21) \times 10^{2}$ e
	1000-2000	ND
Streptomycin	31.25	$(1.25 \pm 0.12) \times 10^{4}$ a
	62.5	$(1.21 \pm 0.10) \times 10^{3}$ a
	125	$(1.01 \pm 0.43) \times 10^{2}$ b
	250-2000	ND

Data are presented as mean ± SD (n = 3). Different letters indicate significant differences at $P < 0.05$.

Table S2. Joint comparison of DAP and MC treatments on the swimming motility diameter of *R. pseudosolanacearum* across equivalent concentrations after 48 h of incubation

Concentration (μg/mL)	Swimming motility diameter		*p*-value	Significance
	DAP	MC		
20	23.53±1.55	18.81±2.16	0.0252	*
40	16.72±1.38	13.92±1.08	0.0143	*
80	9.51±1.09	9.68±0.49	0.6785	ns
160	2.84±0.17	1.46±0.34	0.3055	ns

Note: Data are expressed as mean ± standard deviation (Mean ± SD, n = 3). Pairwise statistical comparisons between DAP and MC at each identical concentration were analyzed using Student's two-tailed independent *t*-test. ns, not significant ($p > 0.05$); *, statistically significant ($p < 0.05$).

Table S3. Joint comparison of DAP and MC treatments on the biofilm formation of *R. pseudosolanacearum* across equivalent concentrations after 36 h of incubation

Concentration (μg/mL)	OD530		*p*-value	Significance
	DAP	MC		
20	0.099±0.0035	0.072±0.0073	0.0247	*
40	0.059±0.0071	0.054±0.0057	0.2918	ns
80	0.043±0.0044	0.018±0.0025	0.0006	**
160	0.018±0.0032	0.003±0.0003	0.0003	**

Note: Data are expressed as mean ± standard deviation (Mean ± SD, n = 3). Pairwise statistical

comparisons between DAP and MC at each identical concentration were analyzed using Student's two-tailed independent *t*-test. ns, not significant ($p > 0.05$); *, statistically significant ($p < 0.05$); **, statistically significant ($p < 0.01$).

Table S4. Joint comparison of DAP and MC treatments on the EPS production of *R. pseudosolanacearum* across equivalent concentrations after 24 h of incubation

Concentration (μg/mL)	EPS production (μmol/g·h)		*p*-value	Significance
	DAP	MC		
20	15.92±1.33	17.91±1.15	0.0125	*
40	9.84±0.76	12.28±0.61	0.0062	**
80	4.53±0.44	6.22±0.55	0.0025	**
160	1.06±0.08	1.01±0.09	0.2362	ns

Note: Data are expressed as mean ± standard deviation (Mean ± SD, n = 3). Pairwise statistical comparisons between DAP and MC at each identical concentration were analyzed using Student's two-tailed independent *t*-test. ns, not significant ($p > 0.05$); *, statistically significant ($p < 0.05$); **, statistically significant ($p < 0.01$).

Table S5. Sequencing and quality control statistics of the *R. pseudosolanacearum* transcriptome.

Sample name	Raw reads	Raw bases	Clean reads	Clean bases	Error rate (%)	Q30 (%)	GC content (%)
MC_1	7857082	1.18G	7634584	1.15G	0.01	96.05	64.63
MC_2	8147572	1.22G	7981648	1.2G	0.01	96.45	64.68
MC_3	7608292	1.14G	7456890	1.12G	0.01	95.94	64.53
DAP_1	7804220	1.17G	7636524	1.15G	0.01	95.89	65.3
DAP_2	8471164	1.27G	8295258	1.24G	0.01	96.86	64.99
DAP_3	8134902	1.22G	7959710	1.19G	0.01	95.95	64.45
CK_1	7904364	1.19G	7698880	1.15G	0.01	96.36	64.4
CK_2	6858650	1.03G	6674496	1.0G	0.01	95.95	64.98
CK_3	7866586	1.18G	7639462	1.15G	0.01	96.25	64.64

Table S6. Differential expression of genes in core metabolic pathways of the *R. pseudosolanacearum* under MC treatment

Significantly Enriched Metabolic Pathway	Gene Symbol
Aminobenzoate degradation	*acyP2*
	acyP1
	bclA
	sdgC
flagellar assembly	*FliE*
	FliF
	FliG
	FliH

	FliI
	FliJ
	FliK
	FliL
	FliM
	FliN
	FlhA
	flgA
	flgB
	flgC
	flgD
	flgE
	flgF
	flgG
	flgH
	flgI
	flgJ
Chemotaxis	*MCP*
	cheW
	cheY
	ChpA
EPS biosynthesis	*XpsR*
	EpsA
	EpsP
	EpsB
	EpsC
	EpsD
	epsF
	epsE
Mineral and organic ion transporter	*cysW*
	SsuA
	malK
	somE
	somF
	somG
	BmpA
	NupC
	RbsB
	RbsA
	AraF
	AraG
	AraH
	XylG

	XylH
	XylF
	IatA
	ugpB
	ugpA
	ugpE
	ugpC
Phenylacetate degradation	*paaK*
	paaA
	paaE
	paaC
	paaD
	paaB
	paaG
	fadB1
	hbd
Plant cell wall-degrading enzymes	*Egl*
	Pme
	CbhA
Quorum_sensing	*anoI*
	appF
	BruAb2_1032
	cqsS
	dppA

Table S7. Differential expression of genes in core metabolic pathways of the *R. pseudosolanacearum* under DAP treatment

Significantly Enriched Metabolic Pathway	Gene Symbol
Aerobic respiration	*ccoN*
	fixN
	ccoO
	cydA
	cydB
Aminobenzoate degradation	*acyP2*
	bclA
	sdgC
	fadB1
EPS biosynthesis	*XpsR*
	epsA
	epsB

	epsC
	epsD
	epsE
	epsF
Folate biosynthesis	*folE2*
	moaA
	GM001504
	queE
	phhA
	phhB
Lipoic acid metabolism	*bkdA*
	pdhB
	bkdB
	Novel00184
	Novel00183
	Novel00185
Phenylacetate degradation	*paaA*
	paaE
	paaD
	paaG
	PaaF
Starch and sucrose metabolism	*PfkB*
	glgA
	malQ
	glgB
	glgE
	treS
	treZ
Tryotophan metabolism	*fadB1*
	gcdH
	kynA1
	kynA
	kynB
	kynU
	Novel00310
Valine, leucine and isoleucine degradation	*acdA*
	bkdA
	bkdB
	fadB1
	Novel00183<br>Novel00184
	Novel00185
	pdhB

	fadD
	flhC
	flhD
	Novel00339
	oppD
	oppF
	XCC0851
TypeIII secretion system	*sctC*
	hrpB3
	GM001324
	GM001311
	hrcV
	hrcU
	sctN
	GM001322
	GM001314
Type VI secretion system	GM003526
	GM003530
	GM003532
	clpV1
	Novel00373
	Novel00374
	tssM1
	vgrG1a
	vgrG1b
	vgrG2b
